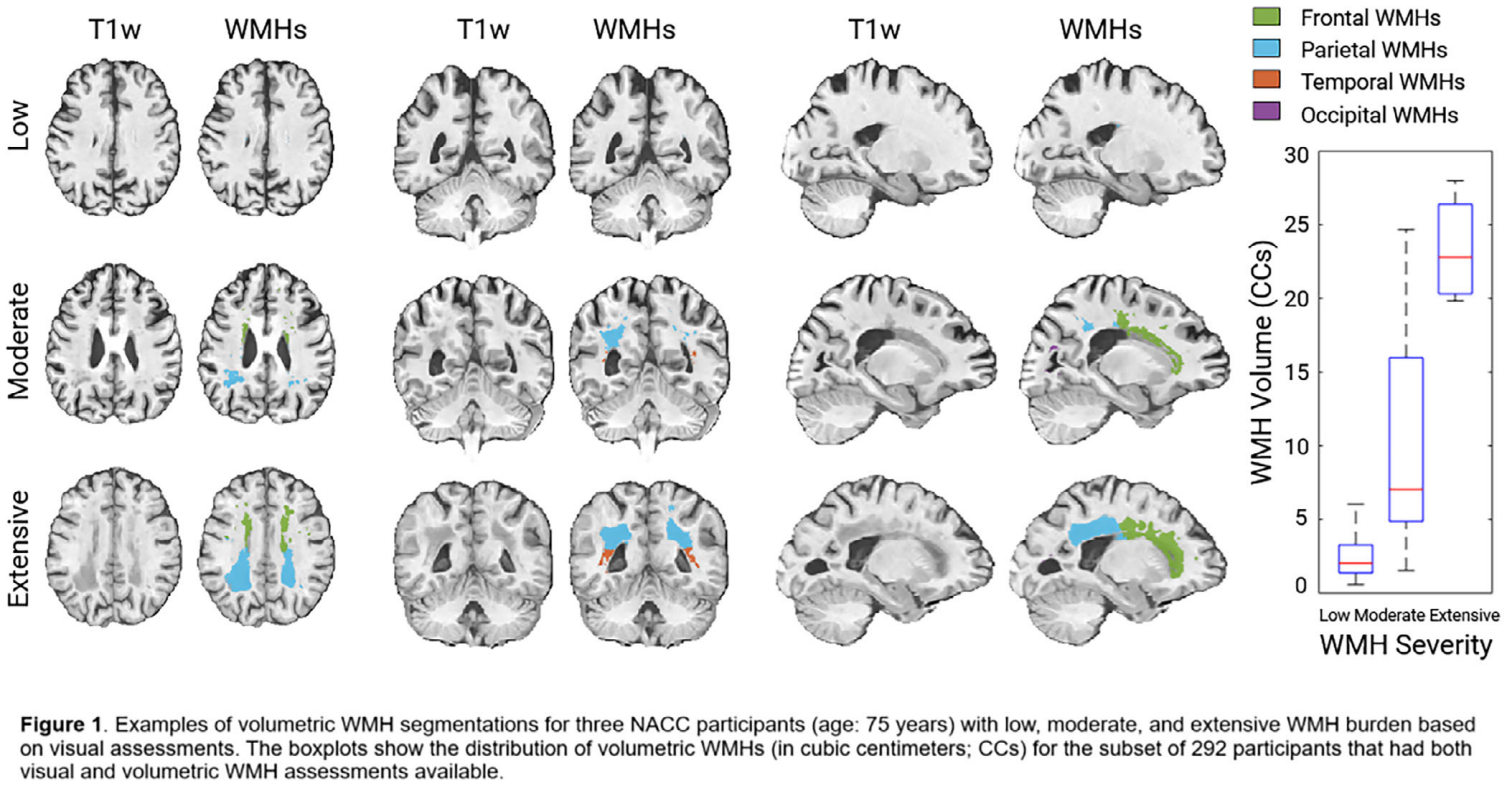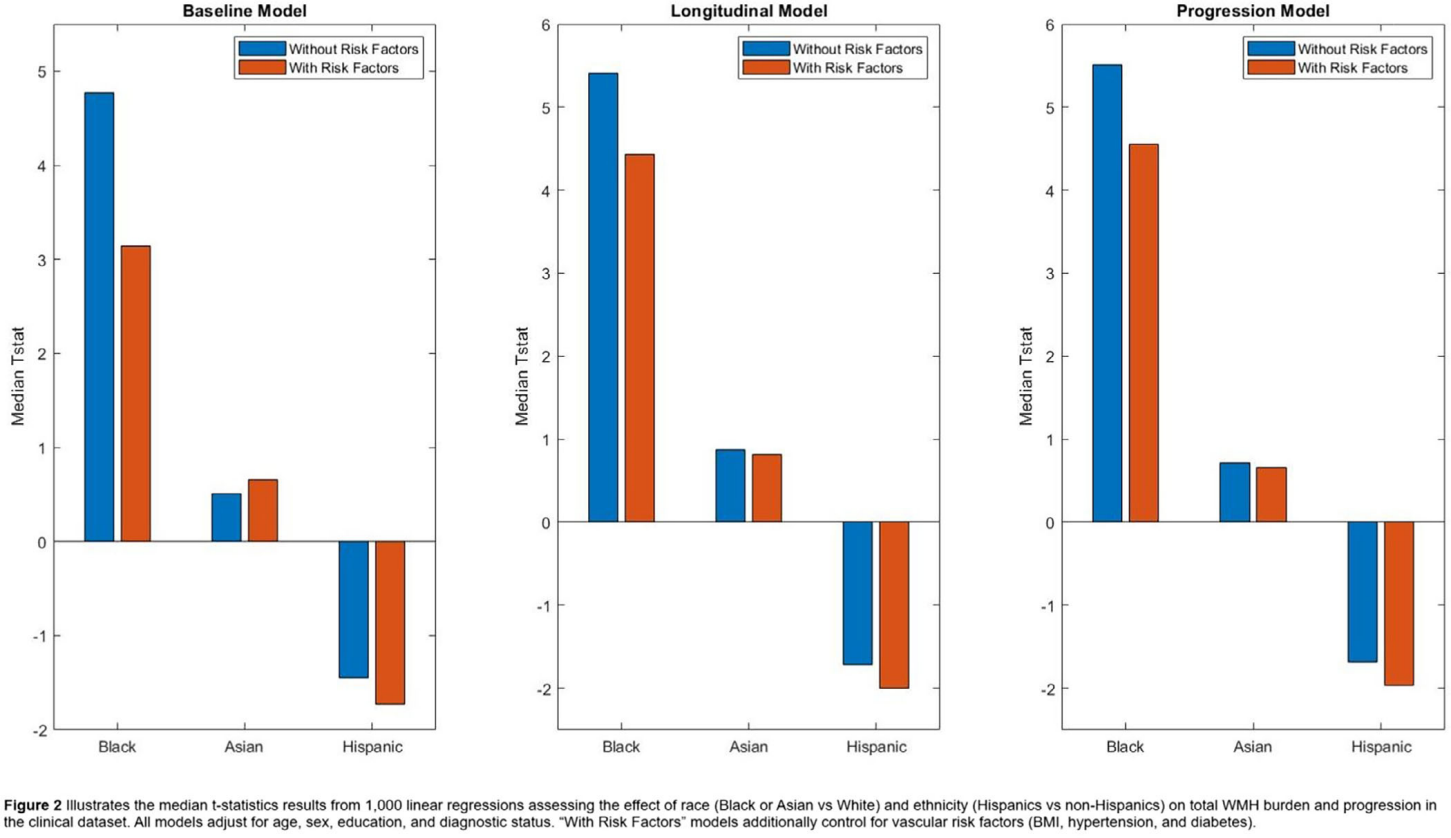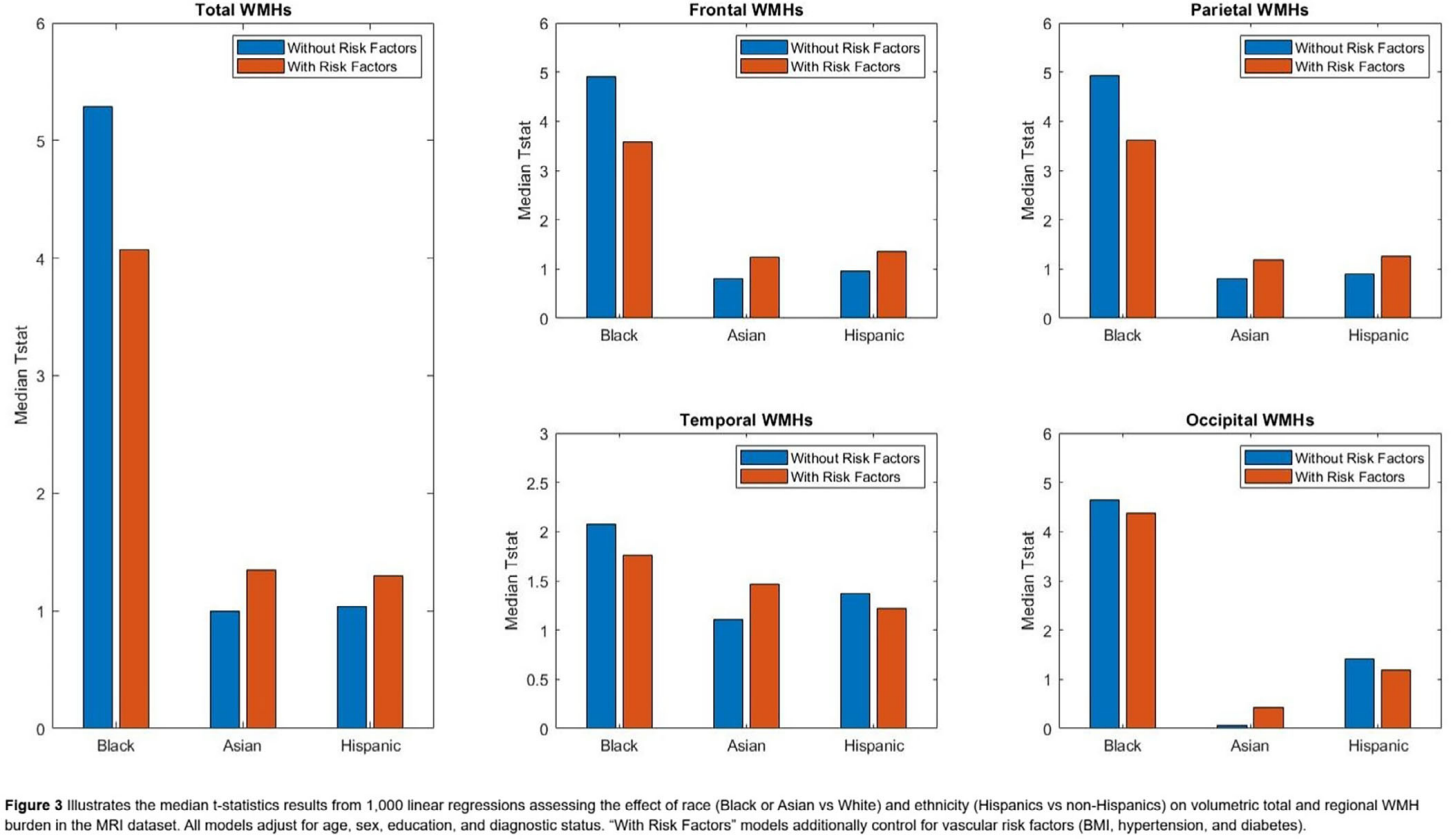# Racial and Ethnic Differences in White Matter Hyperintensity Burden: The Role of Vascular Risk Factors

**DOI:** 10.1002/alz70856_106667

**Published:** 2026-01-08

**Authors:** Farooq Kamal, Roqaie Moqadam, Cassandra Morrison, Mahsa Dadar

**Affiliations:** ^1^ Douglas Mental Health University Institute, McGill University, Montreal, QC, Canada; ^2^ Douglas Mental Health University Institute, Montréal, QC, Canada; ^3^ Carleton University, Ottawa, ON, Canada; ^4^ McGill University, Montréal, QC, Canada

## Abstract

**Background:**

A critical pathological marker observed in the aging brain is white matter hyperintensities (WMHs). WMHs are associated with an increased risk of cognitive decline, progression to mild cognitive impairment, and development of dementia. Vascular risk factors such as hypertension, diabetes, and elevated body mass index are well known contributors to WMH burden and independently increase the risk of dementia. These risk factors are disproportionately prevalent in racially diverse populations. However, the role of vascular risk factors in explaining WMH differences is not well understood in racially and ethnically diverse populations. This study examined whether race and ethnicity influence WMH burden and whether vascular risk factors explain these differences.

**Method:**

Clinical and MRI data from the National Alzheimer's Coordinating Center (NACC) included 7,132 Whites, 892 Blacks, 283 Asians, 8307 non‐Hispanics, and 661 Hispanics. Baseline and longitudinal WMHs (Figure 1) were examined using linear regression and mixed‐effects models across racial and ethnic groups, controlling for demographics and vascular risk factors.

**Result:**

At baseline, significant WMH burden differences were found between Black vs White older adults in total, frontal, temporal, parietal and occipital regions (*t* ranging between 2.07–5.29, *p* <0.001). After adjusting for vascular risk factors, WMH burden differences were reduced in total, frontal, parietal, and occipital regions (*t* ranging between 3.58‐4.37, *p* <0.001) and eliminated in temporal regions (*t* = 1.76, *p* = 0.08). In the longitudinal dataset, when comparing Hispanics to non‐Hispanics, significant differences in total WMH burden were found only after adjusting for vascular risk factors (*t* = 2.00, *p* = 0.04). No significant WMH burden differences were observed between Asian and White participants at baseline or longitudinally (*p*>0.05). See Figure 2 (total WMH volume) and 3 (total and regional WMH volume) for median t‐statistics from the WMH analyses.

**Conclusion:**

Vascular risk factors contribute to some of the racial WMH burden differences between Black and White older adults. The reverse effect was observed in Hispanic vs non‐Hispanic populations, with addition of vascular risk factors revealing ethnic group differences. These findings highlight the importance of exploring how the interaction between risk factors and race/ethnicity contributes to brain changes in the aging population.